# Association Between Rapid Antigen Testing and Antibiotic Use and Accuracy of Peripheral Blood Parameters in Detecting Group A Streptococcus in Children With Tonsillopharyngitis

**DOI:** 10.3389/fped.2019.00322

**Published:** 2019-08-02

**Authors:** Yakup Cag, Abdurrahman Avar Özdemir, Ufuk Yükselmiş, Ezgi Akdeniz, Mustafa Özçetin

**Affiliations:** ^1^Department of Pediatrics, Kartal Dr. Lutfi Kırdar Training and Research Hospital, University of Health Sciences, Istanbul, Turkey; ^2^Department of Pediatrics, Biruni University Medicine Hospital, Istanbul, Turkey; ^3^Department of Pharmacy, Süreyyapaşa Chest Diseases and Thoracic Surgery Training and Research Hospital, University of Health Sciences, Istanbul, Turkey; ^4^Department of Pediatrics, Faculty of Medicine, Istanbul University, Istanbul, Turkey

**Keywords:** bacterial antigens, child, drug prescriptions, immunoenzyme techniques, streptococcal infections, tonsillopharyngitis

## Abstract

**Objectives:** To investigate the effect of rapid antigen testing (RAT) on the practice of antibiotic prescription as well as the accuracy of peripheral blood neutrophil-to-lymphocyte ratio (NLR) and C-reactive protein (CRP) values in detecting group A beta-hemolytic *Streptococcus* (GABHS) in children with tonsillopharyngitis.

**Methods:** In a multicenter study performed in Turkey, we retrospectively analyzed data from 668 consecutive pediatric patients under 17 years of age, who presented with signs and symptoms of tonsillopharyngitis and underwent RAT. The rates of positive and negative RAT results were determined and patients' antibiotic prescriptions were examined in relation to RAT results. In addition, the accuracy of peripheral blood NLR and CRP values was examined for 212 patients whose laboratory data were available, with RAT as the reference standard.

**Results:** Positive RAT results were observed in 190 of 668 (28.4%) patients. Antibiotics were prescribed to all 190 patients with positive RAT results and to 8 of 478 patients with negative RAT results. Overall, the rate of antibiotic prescription was 29.6%. Patients with positive and negative RAT results did not differ significantly with regard to NLR and CRP values. In ROC analysis, the area under the ROC curve (AUC) of NLR and CRP were 0.54 (95% confidence interval [CI] 0.45–0.64), and 0.55 (95% CI 0.45–0.65), respectively.

**Conclusion:** RAT results proved highly associated with antibiotic prescribing, suggesting that RATs could be of great value in preventing unnecessary antibiotic use. Our findings also suggest that NLR and CRP are poorly accurate to identify GABHS in children with tonsillopharyngitis.

## Introduction

Tonsillopharyngitis is a common upper respiratory tract infection (URTI) in children, mainly associated with viruses, manifesting as acute inflammation of the tonsils and pharynx. Bacteria are also responsible for the condition, with *Streptococcus pyogenes*, also known as group A beta-hemolytic *Streptococcus* (GABHS), being the most important bacterial cause (1). In a meta-analysis of studies involving children with sore throat, the pooled prevalence rates of GABHS were 37% and 24% among children of all ages and in those younger than 5 years of age, respectively (2). As one of the leading causes of presentation to health-care facilities worldwide, GABHS tonsillopharyngitis afflicts about 450 million children every year (3, 4). Pediatric population accounts for nearly half of all cases, the majority of which are at ages of 5–15 years (30–37%) and the remaining (5–10%) under 5 years of age (4).

GABHS tonsillopharyngitis may cause early suppurative complications and non-suppurative complications such as acute rheumatic fever and acute glomerulonephritis, requiring early diagnosis and treatment. However, due to overlapping symptoms, its clinical differentiation from viral tonsillopharyngitis may be difficult (5, 6). Although laboratory findings such as neutrophil, lymphocyte and white blood cell (WBC) counts and an elevated C-reactive protein (CRP) level may be suggestive of a bacterial infection, they are not sufficient for the identification of the pathogenic agent (7). Throat culture is the reference standard for diagnosing GABHS pharyngitis, but requires 24-h for identification. Rapid antigen-detection tests (RAT) are easy to perform, providing a rapid diagnosis with a sensitivity of 65–96% and a specificity of >95% (8). Whether GABHS are promptly diagnosed or ruled out is of particular importance in that a positive test result would prompt antibiotic use by indication while a negative test result would deter from antibiotic use. This is not only beneficial to patients by reducing unnecessary antibiotic use, and in turn, adverse drug reactions and antibiotic resistance, but also in favor of the society as a whole in the context of decreasing overall antibiotic resistance and reducing health-care expenditures (8). The use of RATs has been shown to be influential in decreasing the rates of antibiotic prescription (9–11).

The value of the neutrophil-to-lymphocyte ratio (NLR) as an adjunct marker has been extensively studied in the diagnosis of a wide range of disease processes in pediatric patients (12–16). However, to our knowledge, neither the predictive value of the NLR nor its agreement with RAT has been assessed in cases with tonsillopharyngitis.

In this retrospective study, we investigated the association between RAT results for GABHS and antibiotic prescribing in children under 17 years of age, who were diagnosed with URTI and had signs and symptoms of tonsillopharyngitis. Moreover, the association between RAT results and peripheral blood NLR and CRP values was also evaluated. We, therefore, sought to find out how much RAT for GABHS influenced antibiotic prescribing and how much the NLR could predict GABHS in pediatric patients.

## Methods

### Study Design

We performed a multi-center, retrospective cohort study among pediatric patients who were admitted to the outpatient clinics of regional referral hospitals. The study was approved by the Institutional Review Board of Sureyyapasa Chest Diseases and Thoracic Surgery Training and Research Hospital, Istanbul, Turkey (no. 116.2017/024) and was conducted in accordance with the ethical principles of the Declaration of Helsinki. Because of its retrospective design, written informed consent was not required from the parents. The study only retrieved the patients' electronic medical data without identification information. For this retrospective study, written informed patient/parental consent was waived by the Institutional Review Board. The standards for reporting of diagnostic accuracy studies (STARD) checklist 2015 (17) was used for reporting, as shown in Supplementary Material.

### Participants

**Figure 1:**
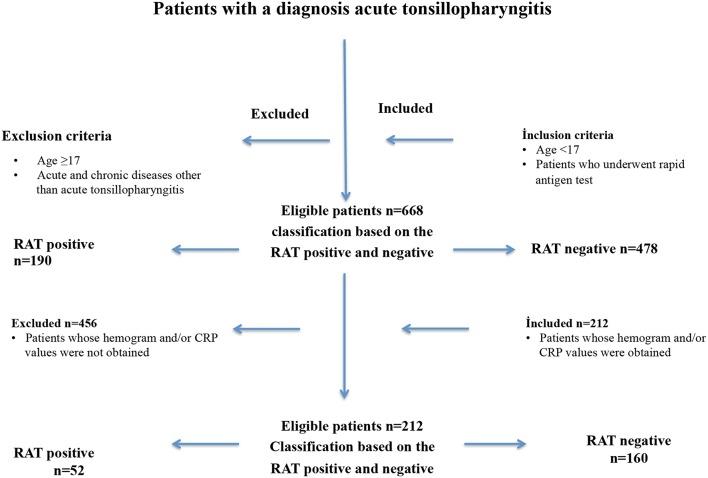
The enrolment and exclusion criteria of pediatric patients (*n* = 668) with acute tonsillopharyngitis who were included in this retrospective observational, cross sectional study.

This study included consecutive pediatric patients who presented between January 1 and September 20, 2017, to a total of 10 health care centers, including three tertiary hospitals and seven community hospitals. The inclusion criteria comprised: children under 17 years of age, who presented with signs and symptoms of tonsillopharyngitis, and who underwent RAT because of a diagnosis of URTI with an initial suspicion of GABHS based on scores of 2 or higher according to the modified Centor criteria (Figure 1 and Table 1) (18). In addition, patients' records were examined to determine whether they had had another visit within 7 days after the initial presentation.

**Table 1:** The modified Centor criteria and scoring.

**Features**	**Score**
Fever (>38.0°C, axillary measurement)	1
Enlarged and tender anterior cervical nodes	1
Tonsillar swelling or exudate	1
No cough	1
Age (years)	
3–14	1
15–44	0
≥45	−1

### Test Methods

In Turkey, through a nationwide training campaign in 2017 for physicians in the context of rational antibiotic use, the use of Strep A Extraction Reagent 1 (Pro-Lab, Austin, US) has been standardized for antigen testing. Accordingly, all patients with a diagnosis of URTI and an initial suspicion of GABHS based on scores of 2 or higher according to the modified Centor criteria underwent RAT for GABHS. Antibiotic therapy was initially prescribed only for those with a positive RAT result.

The RAT technique and interpretation of results were carried out according to the recommendations of the manufacturer. Strep A Extraction Reagent 1 (Pro-Lab, Austin, US) is a qualitative, membrane-based immunochromatographic test for the detection of group A carbohydrate antigen from throat swabs. The test strip is pre-coated by antibodies specific to group A carbohydrate antigen. During testing, exposure of the processed throat swab specimen onto the test strip results in binding of group A carbohydrate antigens to antibodies conjugated to detector particles on the test strip. The antigen-conjugate complex is allowed to migrate across the test strip, and if GABHS are present in the specimen, they react with the antibodies immobilized in the “T” test line. Generation of a colored test line indicates a positive result, while the absence of a colored test line indicates a negative result. The tests were performed in the examination room by the physicians who had previously received a RAT-specific training program.

In addition, data from a convenience subset of patients whose hemogram and/or CRP values were obtained on the day of presentation were retrieved to assess the association between RAT results and peripheral blood NLR and CRP values. To calculate the NLR, the absolute neutrophil count was divided by the absolute lymphocyte count. The ratios of the absolute platelet count (PC) to the mean platelet volume (MPV) (PC/MPV) and platelets to lymphocytes (PLR) were also calculated.

### Statistical Analysis

Demographic characteristics (age, sex), RAT results, hemogram and CRP values of the patients were analyzed. Variables with normal distribution (age, hemogram values) were expressed as mean and standard deviation, while non-normally distributed variables were expressed as median and interquartile range (IQR).

The rates of positive and negative RAT results were determined and patients' antibiotic prescriptions were examined in relation to the RAT results. Further analysis to test the accuracy of the RAT with reference to throat cultures was not possible because of the small number of cases with throat cultures.

Between-group differences based on the positive and negative RAT results were analyzed with the chi-square test for dichotomous variables, with the Student's *t*-test for normally distributed variables, and with the Mann–Whitney *U*-test for non-normally distributed variables. The diagnostic accuracies of NLR, PLR, PC/MPV, and CRP were also assessed by a receiver operating characteristic (ROC) curve analysis for each variable. The result of the RAT was used as the reference standard for ROC analysis. A *P* < 0.05 was considered statistically significant. There was no specific sample size calculation for this study.

## Results

### Participants

Of 668 included pediatric patients [51% males; median [IQR] age 7 (5–11) years] who underwent RAT, 190 (28.4%) had positive RAT results, for which antibiotic treatment was initiated. The median (IQR) age of the 212 patients (54% males) whose hemogram and/or CRP values were obtained at presentation was 6 (4–9) years. Among these 212 patients, 52 (24.5%) had positive RAT results (Figure 1). Demographic characteristics and hemogram values of patients with positive and negative RAT results are summarized in Table 2. No adverse event occurred associated with the RAT.

**Table 2:** Demographic characteristics and hemogram values of 212 patients with positive or negative rapid antigen test (RAT) results^*^.

	**RAT (–) group**	**RAT (+) group**	***P***
*N* (%)	160 (75.5)	52 (24.5)	
Sex, males *N* (%)	86 (53.8)	28 (53.8)	0.99
Age, median (IQR) (years)	6.0 (4–9)	6.0 (4–9)	0.75
Leukocyte count (× 10^9^/L)	9.2 (7.3–12.0)	9.5 (7.4–12.8)	0.40
Neutrophil count (× 10^9^/L)	4.5 (3.15–7.45)	5.0 (3.9–8.0)	0.29
Lymphocyte count (× 10^9^/L)	2.85 (2.2–3.8)	2.7 (2.1–3.8)	0.53
Monocyte count (× 10^9^/L)	0.7 (0.6–1.05)	0.7 (0.6–1.1)	0.44
Eosinophil count (× 10^9^/L)	0.1 (0.1–0.3)	0.1 (0.0–0.2)	0.67
Basophil count (× 10^9^/L)	0 (0–0.1)	0 (0–0.1)	0.55
Erythrocyte count (× 10^9^/L)	4.7 (4.5–5.0)	4.7 (4.4–4.9)	0.23
Hemoglobin (g/dl)	12.4 (11.8–13.0)	12.5 (11.4–13.0)	0.94
Hematocrit (%)	37.3 (35.3–39.1)	37.0 (34.0–38.5)	0.45
MCV (fL)	78.4 (75.9–81.1)	78 (75.3–82.2)	0.64
RDW (%)	13.7 (13.1–14.6)	13.3 (12.7–14,.2)	0.0021
Platelet count (× 10?/L)	299.5 (250–373.5)	300.5 (258–357.5)	0.83
MPV (fL)	7.9 (7.4–8.5)	7.8 (7.2–8.7)	0.99
PDW (fL)	16.4 (16.0–16.7)	16.4 (15.9–16.6)	0.30

* *All hemogram values are expressed as median and interquartile range (IQR); MCV, mean cell volume; RDW, red cell distribution width; MPV, mean platelet volume; PDW, platelet distribution width*.

### Association Between RAT Results and Antibiotic Use

Antibiotics were prescribed to all 190 patients who had a positive RAT result. Within 7 days after the initial presentation, 44 patients (6.6%), who all had had negative RAT results, were found to have been seen at control visits within. Of these, 21 patients received a diagnosis of URTI, of whom only 8 patients were then placed on antibiotic treatment, but without a repeat RAT. The remaining patients had diagnoses other than URTI and did not receive antibiotic treatment. The overall antibiotic use was 29.6% (198/668).

### Accuracy of Peripheral Blood Parameters

Patients with positive and negative RAT results had similar blood count parameters except for the red cell distribution width between patients with positive and negative RAT results. The between-group differences in the NLR, PLR, PC/MPV, and CRP were not significant between the positive and negative RAT groups (Table 3). In ROC analysis, the area under the ROC curve (AUC) for NLR and CRP were 0.54 (95% confidence interval [CI] 0.45–0.64], and 0.55 (95% CI 0.45–0.65), respectively (Table 4).

**Table 3:** NLR, PLR, PC/MPV, and CRP values of 212 patients with positive or negative rapid antigen test (RAT) results.

	**RAT (–) group**		**RAT (+) group**		
	***N***	**Median (IQR)**	***N***	**Median (IQR)**	***P***
NLR	160	1.60 (0.86–2.94)	52	1.86 (0.99–3.40)	0.29
PLR	160	107.64 (79.33–142.50)	52	110.36 (83.90–134.17)	0.97
PC/MPV	159	38.25 (30.43–48.90)	51	36.14 (27.95–45.27)	0.45
CRP, mg/dL	78	3.44 (0.37–17.80)	20	4.39 (0.76–35.35)	0.23

*NLR, neutrophil-to-lymphocyte ratio; PLR, platelet-to-lymphocyte ratio; PC/MPV, platelet count-to-mean platelet volume ratio; CRP, C-reactive protein; IQR, interquartile range*.

**Table 4:** ROC analysis for the diagnostic accuracy of NLR, PLR, PC/MPV, and CRP in detecting group A Streptococcus in children with tonsillopharyngitis.

	***N***	**Area under ROC curve**	**%95 confidence interval**
NLR	212	0.54	0.45–0.64
PLR	212	0.54	0.45–0.63
CRP	98	0.55	0.45–0.65
PC/MPV	210	0.50	0.41–0.59

*NLR, neutrophil-to-lymphocyte ratio; PLR, platelet-to-lymphocyte ratio; PC/MPV, platelet count-to-mean platelet volume ratio; CRP, C-reactive protein; ROC, receiver operating characteristic*.

## Discussion

Our study indicated that the use of RAT for GABHS could reduce the practice of antibiotic prescription. Furthermore, neither between-group differences nor ROC analysis showed a diagnostic value of NLR and CRP to diagnose GABHS tonsillopharyngitis.

In cases of GABHS tonsillopharyngitis, antibiotic treatment is essential for the eradication of GABHS, thus preventing further complications and reducing the transmission rate of GABHS. Conversely, in cases where the diagnosis of GABHS cannot be ruled out by reliable methods, unnecessary antimicrobial therapy may be an inappropriate choice, contributing to the growing public health problem of antibiotic resistance (8). According to the Centers for Disease Control and Prevention (CDC), more than 2 million persons in the US each year become infected with bacteria resistant to antibiotics, leading to at least 23,000 deaths each year as a direct result of these infections (19). Even more alarming is the 2015 CDC report on outpatient antibiotic prescriptions, with 838 antibiotic prescriptions per 1,000 persons per year (20). In a 2014 report by Versporten et al. Turkey was found to have the most frequent use of antibiotics across 13 non-European countries (21). Van Boeckel et al. assessed antibiotic consumption based on sales data between 2000 and 2010 for 71 countries and reported an increase by 35% (22). Several studies reported high antibiotic prescription rates between 53 and 72% for patients diagnosed with URTI (11, 23).

Highly overlapping clinical manifestations of viral and GABHS tonsillopharyngitis pose a challenge to the diagnosis and treatment of affected patients (5). In a study from Brazil, petechiae, purulent exudate and painful tonsils were more frequent in children with a positive GABHS culture. However, the diagnostic accuracy of clinical signs was found to be low (24). The sensitivity and specificity rates of RATs have been shown to vary between 58 and 96% and between 92 and 97%, respectively (1, 8, 25). The RAT has a high potential to show GABHS-positive patients; hence the need to treat with antibiotics. In case of a negative RAT result, the chance of missing a GABHS infection would be <5%, or 1 in 20 patients (26). As an immediate and reliable method of identification, RAT is thought to contribute to a more rational utilization of antibiotics in tonsillopharyngitis, thus preventing excessive and inappropriate antibiotic use. In a study involving 650 children and adolescents, the rate of actual antibiotic prescription was 44% based on RAT results as compared with a presumable rate of 59.8% based on clinical findings (9). Of note, among the children who would not have received antibiotics based on the clinical evaluation, 42.5% turned out to have positive RAT results (9). Indeed, given that empirical antibiotic prescription based on clinical criteria is associated with high rates of antibiotic consumption, RAT may play a pivotal role in decreasing actual antibiotic prescription rates by 41–61% (12, 27). The present study showed an overall antibiotic prescription rate of only 29.6% among 668 pediatric patients who underwent RAT. In Turkey, according to the 2015 data from the Ministry of Health the rates of antibiotic prescribing by physicians for patients diagnosed with tonsillopharyngitis were 61 and 66% in the age groups of 0–3 and 4–17 years, respectively. The corresponding rates were 62 and 68% for 2014 (28). Comparison of our data with the data of 2014 and 2015 shows a clear potential for decrease of approximately 50% in antibiotic prescription rates among children with tonsillopharyngitis.

According to the modified Centor criteria, the risk for GABHS shows a linear relationship with the scores, being 11–17% for score 2, 28–35% for score 3, and 51–53% for score 4 or higher (29). In the present study, all patients with scores of 2 or higher underwent RAT, of whom 28.4% had positive results.

Laboratory findings such as neutrophil, lymphocyte and WBC counts, and acute phase reactants such as CRP are routinely used for the detection of bacterial infections (7). However, these data do not allow to make a clear distinction between bacterial and viral infections, nor to identify GABHS as the causative agent of a possible bacterial infection. Recently, growing interest has been focused on the NLR as an inflammatory marker. Li et al. demonstrated a highly predictive role of the NLR in diagnosis, differentiation, and even prognosis of pulmonary bacterial infections in the elderly (30). In another study in which adult patients were evaluated after admission to the emergency department with suspected community-acquired bacteremia, both lymphocytopenia and the NLR were found to be better predictors of bacteremia than WBC and neutrophil counts and CRP (31). Similarly, the NLR was also shown to be a more useful diagnostic tool to identify patients with septicemia than other more commonly used diagnostic blood tests such as WBC and neutrophil counts and CRP (32).

To our knowledge, the relationship between the NLR and RAT has not been evaluated adequately in cases with tonsillopharyngitis. A recent study from Turkey involving 150 children found significantly higher WBC, NLR, and CRP in GABHS-positive pediatric patients; however, the sample size was relatively small, with culture-positive and rapid test-positive GABHS rates being 7.3 and 9.2%, respectively (33). A study from Denmark investigated 100 patients 15–40 years of age who had acute tonsillitis and found significantly elevated mean values of CRP, WBC, and absolute neutrophil count in patients with GABHS compared with patients without GABHS (34). In the present study, patients with positive and negative RAT results for GABHS tonsillopharyngitis did not differ significantly with regard to the NLR and CRP.

This current study had a number of limitations. Firstly, due to its retrospective design it lacks confirmation of RAT results with throat cultures, the reference standard for diagnosis. Secondly, the reason for blood sampling in a subset of children was unknown. Thirdly, it was not possible to compare signs and symptoms across the groups, because of the lack of clinical information due to a retrospective design. Fourthly, very few patients returned to follow up visits. Fifthly, there was no specific sample size calculation for this study. Finally, the intention of antibiotics prescription by the physician based only on clinical data is lacking.

## Conclusion

As a rapid and reliable method for the identification of GABHS tonsillopharyngitis, RAT proved highly associated with antibiotic prescribing, suggesting that RAT could have a great role in preventing unnecessary antibiotic use. Our findings also suggest that NLR and CRP are poorly accurate to identify GABHS in pediatric patients presenting with signs and symptoms of tonsillopharyngitis.

## Data Availability

All datasets generated for this study are included in the manuscript/Supplementary Files.

## Author Contributions

YC: conceptualization, data curation, formal analysis, investigation, methodology, project administration, resources, software, supervision, validation, visualization, writing—original draft, writing—review and editing. AÖ, UY, EA, and MÖ: data curation, acquisition, investigation, resources, validation, and visualization.

### Conflict of Interest Statement

The authors declare that the research was conducted in the absence of any commercial or financial relationships that could be construed as a potential conflict of interest.
